# In situ transduction of cells in human corneal limbus using adeno-associated viruses: an ex vivo study

**DOI:** 10.1038/s41598-022-26926-0

**Published:** 2022-12-28

**Authors:** Hyeck-Soo Son, Albert S. Jun, James W. Foster, Wei Wang, Yassine Daoud, Gerd U. Auffarth, Madhuparna Roy

**Affiliations:** 1 Wilmer Eye Institute, Johns Hopkins Medical Institutions, 400 North Broadway, Baltimore, MD 21287 USA; 2 University Hospital Heidelberg, Heidelberg, Baden-Wuerttemberg Germany

**Keywords:** Genetics research, Stem-cell research, Corneal diseases

## Abstract

This study aimed to evaluate the efficacy of in situ adeno-associated virus (AAV)-mediated gene delivery into the human corneal limbal region via targeted sub-limbal injection technique. Human cadaveric corneal tissues were fixed on an artificial anterior chamber. Feasibility of sub-limbal injection technique was tested using trypan blue and black India ink. An enhanced green fluorescent protein (eGFP) encoding AAV DJ was injected into sub-limbal region. After AAV injection, corneal tissues were incubated in air-lift culture and prepared for immunohistochemical analysis. Cell survivial and expression of eGFP, stem cell markers (p63α and cytokeratin 19 (KRT19)), and differentiation marker cytokeratin 3 (KRT3) were evaluated using confocal microscopy. Both trypan blue and black India ink stained and were retained sub-limbally establishing specificity of the injection technique. Immunohistochemical analysis of corneas injected with AAV DJ-eGFP indicated that AAV-transduced cells in the limbal region co-express eGFP, p63α, and KRT19 and that these transduced cells were capable of differentiating to KRT3 postitive corneal epithelial cells. Our sub-limbal injection technique can target cells in the human limbus in a reproducible and efficient manner. Thus, we demonstrate that in situ injection of corneal limbus may provide a feasible mode of genetic therapy for corneal disorders with an epithelial etiology.

## Introduction

Inherited corneal disorders such as transforming growth factor beta-inducible (*TGFBI*) dystrophies can cause considerable visual impairment^1–4^. While phototherapeutic keratectomy or partial- and full-thickness corneal transplantation may provide improved vision, the effects are limited as causative mutations remain unaltered in the non-transplanted limbal stem cells (LSCs), and the pathology typically recurs^5^. The global shortage of donor corneas and diminishing returns of repeated surgery further highlights the pressing need for an alternative management modality.

Gene therapies may provide an alternative and potentially permanent treatment to correct the defective or missing transcript. Previous studies using clustered regularly interspaced short palindromic repeat/Cas9 (CRISPR-Cas9)-mediated genome editing technology have shown promising results in targeting the pathogenic *TGFBI* mutations in isolated corneal keratocytes and epithelial stem cells^6,7^. However, to our knowledge, in situ genetic modification of these cells has not been demonstrated.

In recent years, adeno-associated viruses (AAVs) have emerged as vectors of choice for ocular gene therapy thanks to their established clinical efficacy, biosafety profile, and low immunogenicity^8–10^. However, it is not yet known whether AAVs can successfully transduce corneal LSCs. Although intrastromal or intracameral injections have been used for delivery of gene therapy to subepithelial corneal layers, a limbal stem cell modification in situ has not been attempted.

In this study, we investigated targeted sub-limbal injection using ex vivo human corneal tissues. Using trypan blue and black India ink dye as visual guides, we describe this injection technique as a reproducible and feasible method to deliver therapeutic payload directly to cells in limbal region in situ. We also demonstrate that this technique is applicable to AAV-enhanced green fluorescent protein (eGFP) delivery into cells present within the human limbus.

## Results

### Trypan blue and black India ink injections

**Figure 1:**
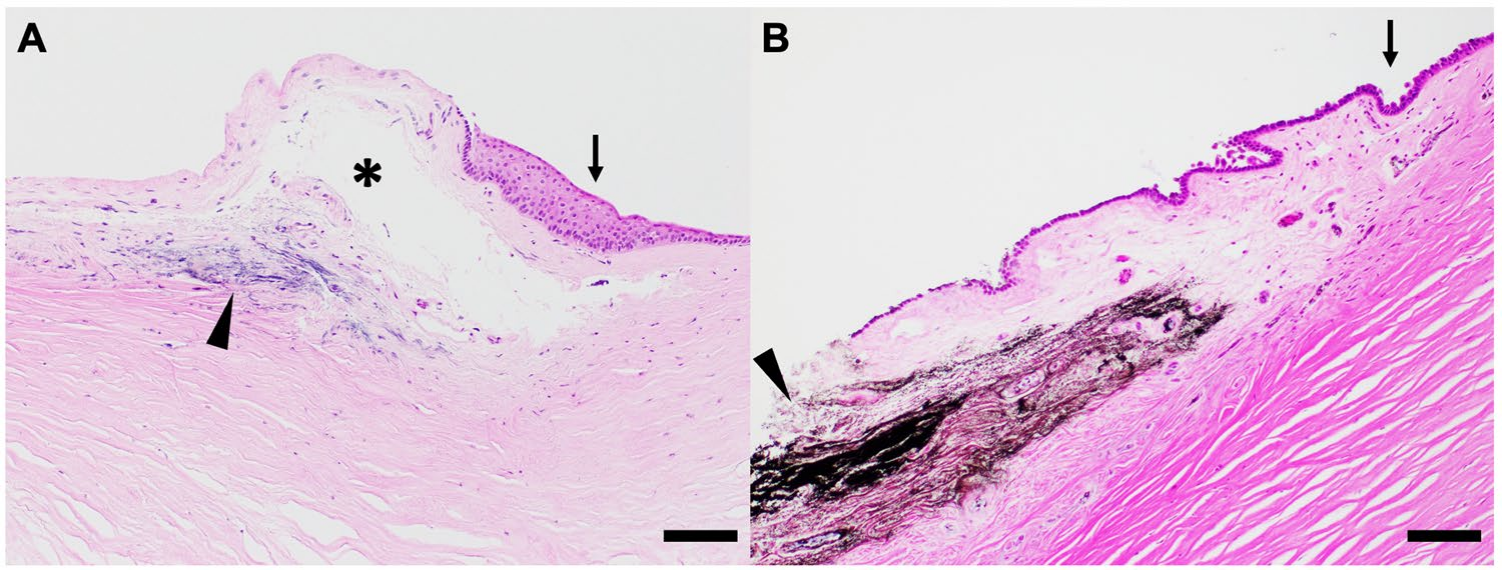
Histological H&E images of the cornea showing trypan blue (**A**) and black India ink (**B**) staining of sub-limbal region after the injection (10 × magnification). A needle track can be seen sub-limbally (**A**, asterisk). Arrowheads point to the injected dye and the arrows indicate corneal limbal cells. Scale bars indicate 200 µm.

Hematoxylin and eosin (H&E) staining demonstrates that both trypan blue (Fig. 1A) and black India ink (Fig. 1B) dyes can be delivered to the sub-limbal region using our injection technique and retained one day after injection. A needle track (Fig. 1A, asterisk) is visible under the limbal region.

### AAV DJ-eGFP injections

#### Day 4

**Figure 2:**
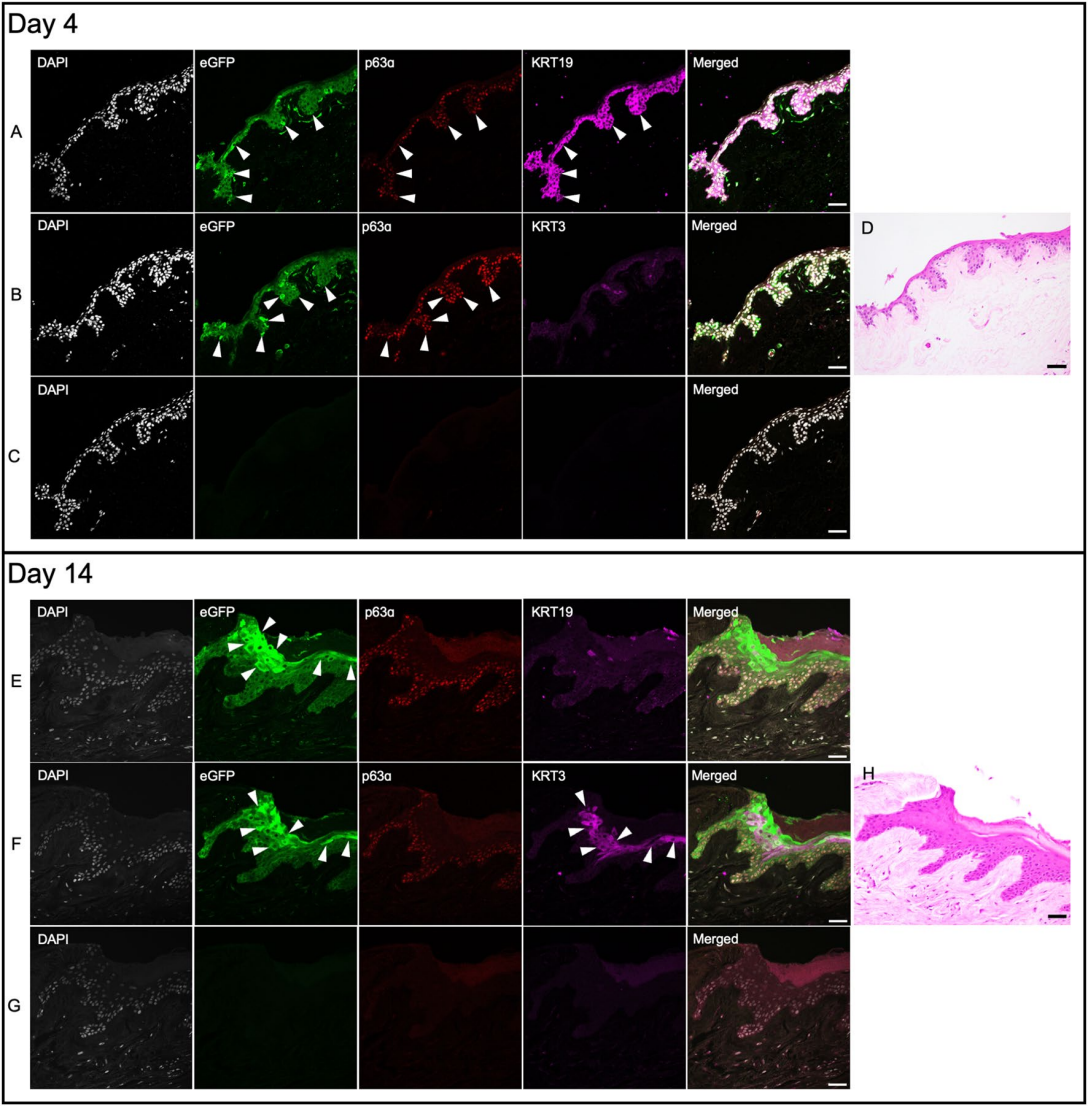
Corneal limbus after four and 14 days of culture show eGFP + cells in situ. Panels (**A**), (**B**) and (**C**) represent three different sections from the cornea that was cultured for four days. (**A**) Stem cell markers p63α (red) and KRT19 (magenta) co-localize with eGFP signal (green) in cells with successful transduction (arrowheads). (**B**) Scant expression of differentiation marker KRT3 (magenta) in eGFP + /p63α + cells (arrowheads). (**C**) Non-primary control confirms the specificity of primary antibody expression. DAPI stained nuclei are shown in gray. (**D**) H&E image demonstrates where the confocal images were taken. Panels (**E**), (**F**) and (**G**) represent three different sections from the cornea cultured for 14 days. (**E**) After 14 days, the eGFP signal is stronger in upper limbal layers (arrowheads) where it no longer co-localizes with stem cell markers p63α and KRT19. (**F**) The eGFP + cells in upper limbal layers co-express differentiation marker KRT3 (arrowheads). (**G**) Non-primary control confirms the specificity of primary antibody expression. (**H**) H&E image demonstrates where the confocal images were taken. All images were captured in 20× magnification and the scale bar indicates 50 µm.

**Figure 3:**
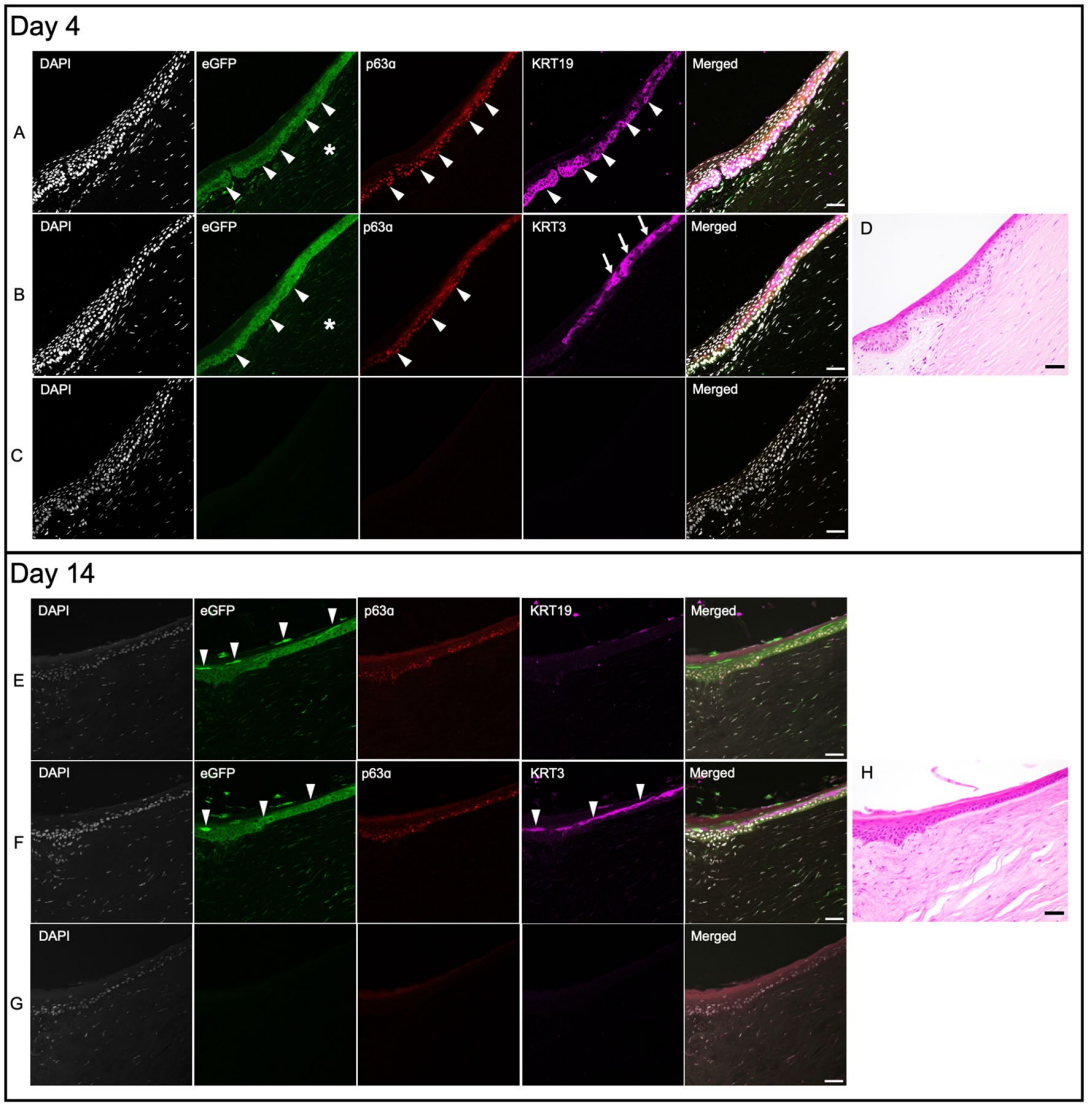
Peri-limbal region after four and 14 days of culture. Panels (**A**), (**B**) and (**C**) represent three different sections from the cornea that was cultured for four days. (**A**) Strong eGFP signal can be seen in peri-limbal corneal epithelium that co-localizes with stem cell markers p63α and KRT19 (arrowheads). eGFP expression can also be observed in stromal cells underneath the limbus (asterisk) resulting from sub-limbal injection. (**B**) eGFP + /p63α + cells are markedly visible in basal layers of peri-limbal epithelium (arrowheads), while differentiation marker KRT3 signal becomes stronger in superficial epithelial layers towards the central cornea (arrows). (**C**) Non-primary control confirms the specificity of primary antibody expression. DAPI stained nuclei are shown in gray. (**D**) H&E image demonstrates where the confocal images were taken. Panels (**E**), (**F**) and (**G**) represent three different sections from the cornea cultured for 14 days. (**E**) Stronger eGFP signal is visible in superficial epithelial layers (arrowheads). (**F**) eGFP + cells co-express KRT3 (arrowheads), suggesting their differentiated nature. (**G**) Non-primary control confirms the specificity of primary antibody expression. (**H**) H&E image demonstrates where the confocal images were taken. All images were captured in 20× magnification, and the scale bar indicates 50 µm.

**Figure 4:**
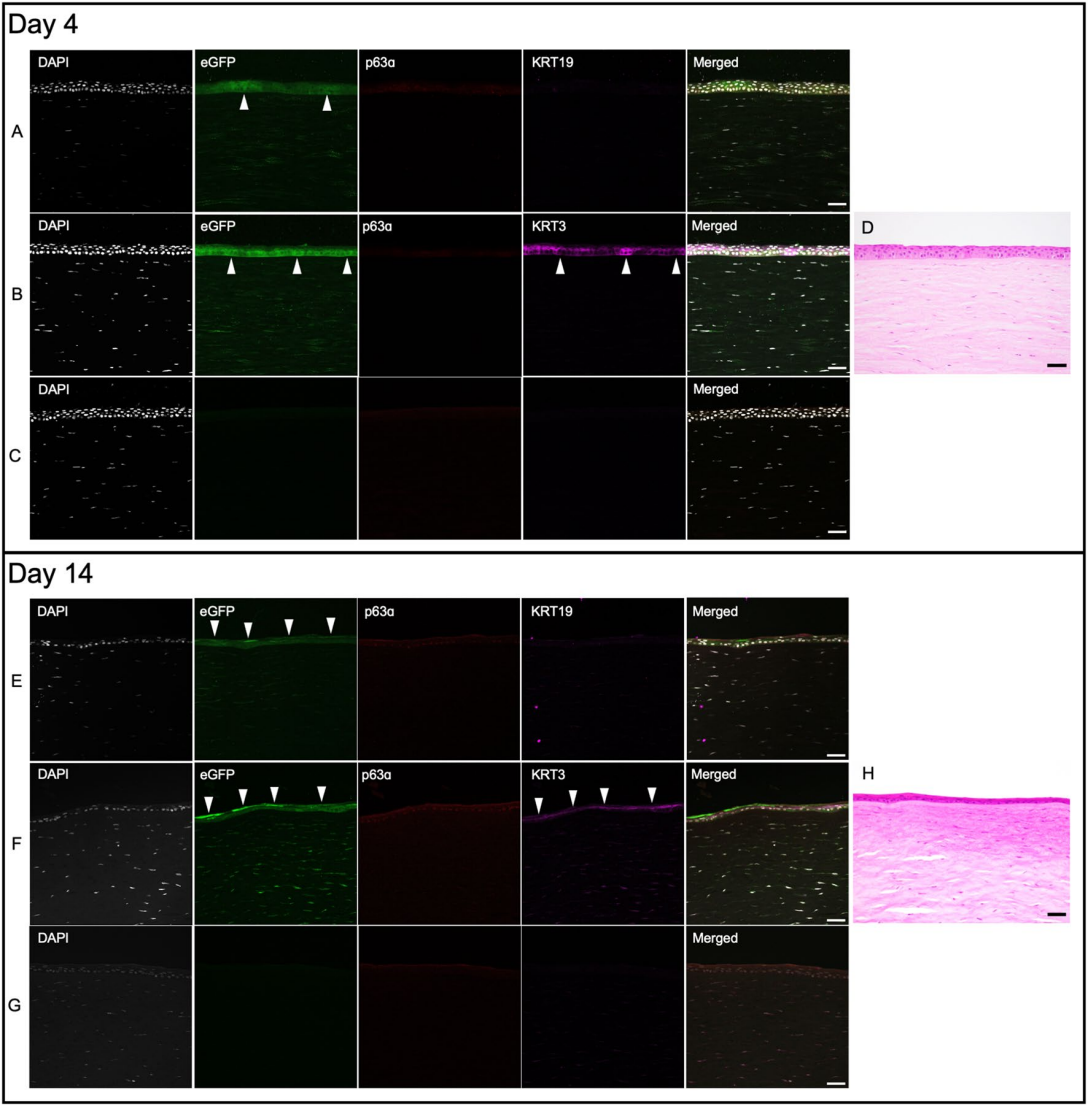
Central cornea after four and 14 days of culture. Panels (**A**), (**B**), and (**C**) represent three different sections from the cornea cultured for four days. (**A**) Compared to limbal or peri-limbal region, eGFP expression is less pronounced in central cornea (arrowheads). The lack of p63α and KRT19 expression suggests the differentiated nature of this area. (**B**) eGFP expression can be seen in basal layers of central epithelium that co-localizes with KRT3 expression (arrowheads). (**C**) Non-primary control confirms the specificity of primary antibody expression. DAPI stained nuclei are shown in gray. **(D)** H&E image demonstrates where the confocal images were taken. Panels (**E**), (**F**), and (**G**) represent three different sections from the cornea that was cultured for 14 days. (**E**) eGFP + cells can be seen in predominantly superficial layers of the epithelium (arrowheads). (**F**) Co-expression of KRT3 in eGFP + cells suggests their differentiated state (arrowheads). (**G**) Non-primary control confirms the specificity of primary antibody expression. (**H**) H&E image demonstrates where the confocal images were taken. Scale bar indicates 50 µm. All images were taken in 20× magnification.

Immunohistochemical analysis of corneas cultured for 4 days show eGFP positive (+) cells (Fig. 2, upper panel, arrowheads) in limbal region which also express stem cell markers p63α and cytokeratin 19 (KRT19) (Fig. 2A), demonstrating successful in situ transduction of cells in human corneal limbus with AAV serotype DJ. Scant expression of differentiation marker cytokeratin 3 (KRT3) suggest the undifferentiated nature of the cells in this region (Fig. 2B). We also observed strong eGFP signal in epithelial cells of peri-limbal region (Fig. 3, upper panel, arrowheads), which tended to gradually attenuate towards the central cornea (Fig. 4, upper panel, arrowheads).

#### Day 14

eGFP + cells could also be identified in limbal region after 14 days of culture (Fig. 2, lower panel, arrowheads). Compared to day 4, we observed stronger eGFP signal in upper layers of limbus, which showed high KRT3 expression (Fig. 2E). Furthermore, analysis of peri-limbal area (Fig. 3, lower panel, arrowheads) and central cornea (Fig. 4, lower panel, arrowheads) also show expression of eGFP + /KRT3 + cells in superficial layers of the epithelium.

## Discussion

In this study, we demonstrate that cells in human corneal limbal compartment can be targeted in a reproducible manner using our sub-limbal injection technique. More importantly, we show that these cells can be transduced successfully in situ using AAVs, highlighting that an injection technique may present a viable delivery approach for viral gene therapy.

Evidence gathered from over 20 years of research attest to the safety and efficacy of AAV gene therapy^11^. Today, two AAV-based gene therapies have been approved by the United States Food and Drug Administration (FDA), while numerous more clinical trials are underway^12^. In ophthalmology, the primary route of AAV vector administration involves intravitreal or subretinal injection for transduction of retinal cells^12,13^. Despite the direct accessibility and immune-privileged state of cornea, only limited data exists on the feasibility of AAV-mediated therapy for human LSCs.

LSCs are mainly responsible for replenishment the corneal epithelium and prevention of conjunctival epithelial cell migration onto the cornea^14^. In patients with corneal dystrophies, the epithelial surface becomes dysfunctional due to absent or non-functioning proteins^15^. Contrary to currently available surgical treatment modalities, gene therapy has the potential to biologically correct the root cause of the condition and offer a permanent solution.

Several groups have attempted to transduce corneal epithelial, stromal, or endothelial cells using AAVs^16–19^. However, to date, there is only one study that investigated the transduction efficiency of human LSCs using AAVs. Song et al. added eight different self-complementary AAV serotypes (AAVs 1–9) harboring GFP reporter to human limbal epithelial cells that were isolated in vitro and observed that AAV 6 demonstrated the highest GFP transgene expression at 24 h post AAV-exposure, followed by AAV 4^20^. Notably, the authors also performed intrastromal injection of AAV 6 in central ex vivo corneas to test the transduction efficiency of LSCs, a technique which has been shown to be feasible in mice corneas^21^. However, after a single injection of 10^11^ vg AAV 6 into the central stroma, the authors did not observe any transduction of human LSCs^20^.

Stem cells are generally known for their resistance to viral transduction. While the exact reason for this remains unknown, they may have evolved a defensive mechanism to protect themselves from genomic manipulation^22,23^.

In our study, we used AAV DJ to transduce cells in human limbal compartment. AAV DJ is a synthetic serotype that contains chimeric capsids of AAV serotypes 2, 8, and 9^24^. It is characterized by high in vitro efficiency, low in vivo biodistribution, high liver affinity, as well as the ability to evade immune neutralization^24^. Consequently, AAV DJ has been used as the lead viral vector in recent gene therapeutic studies^25,26^.

While AAV DJ has also been shown to be efficient in transducing human mesenchymal stem cells in vitro^27,28^, our study is the first to test the transduction efficacy of AAV DJ serotype in cells of human corneal limbal region.

In this study, we first assessed for eGFP transgene expression on day 4 of the experiment, i.e. one day after the third AAV DJ-eGFP injection, to identify any early signs of transduction. We observed positive eGFP signal in all layers of the corneal limbus including the basal layer. The co-expression of stem cell markers p63α and KRT19, as well as the scant expression of differentiation marker KRT3 in these eGFP + cells suggest their initial undifferentiated nature. Crucially, we did not observe any disruption of the epithelial cell layers, suggesting that our technique did not evoke extensive cytotoxicity.

After establishing that our injection technique can transduce cells in the limbal region with AAV DJ, we cultured the intact corneas for subsequent 14 days to observe centripetal migration and differentiation of epithelial cells from the limbal compartment to the central cornea.

After 14 days of culture, eGFP + cells were present in both basal and suprabasilar layers of the limbus. KRT3 expression was stronger in the suprabasilar eGFP + cells compared to p63α and KRT19 signals, suggesting these cells to be transient amplifying cells (TACs) that were basal at the time of AAV DJ-eGFP injection and have now begun to migrate and differentiate in line with the XYZ hypothesis^29^. Peri-limbal and central areas of corneas at day 14 showed eGFP + cells in predominantly upper layers of the epithelium. As corneal epithelium is estimated to undergo a turnover every 7–14 days^30–32^, the eGFP + cells that we observed in basal epithelium at day 4 have now likely migrated superiorly to become upper epithelial layers. However, it is unclear whether a similar turnover time can be expected in limbal and epithelial cells of post-surgical human cadaveric corneas where their integrity may have been compromised.

This proof-of-concept study demonstrates the tolerated and reliable delivery of GFP to human basal epithelial cells in situ. We observed expression in all injection sites (8 per cornea) once viral load had been optimized (data not shown).

Although it is unclear whether eGFP + cells in limbus are actual corneal LSCs, the identification of eGFP, p63α, and KRT19 positive cells at day 4, and KRT3 positive cells after 2 weeks of culture allows us to hypothesize that we may have transduced at least primitive cells in limbal region at time of AAV DJ-eGFP injection. Future investigations will establish the persistence of the signal through larger scale experiments and assess clonogenic expansion^33^. This will enable us to identify whether we have transduced the limbal crypt resident LSCs required for long-duration therapeutic potential. Furthermore, these experiments will enable us to optimize gene delivery efficiency in terms of location and viral payload.

Despite the successful in situ transduction of cells in human corneal limbal region in our ex vivo model, the potential side effects of AAV-mediated gene therapy in human limbus still need to be evaluated, particularly regarding the in vivo immune response, the long-term cell viability following sub-limbal injection, and the effects of non-target tissue transduction^34^. In our study, we observed growth and maintenance of stratified corneal epithelium up to 14 days after AAV application on the first three days, which in our view serves as a confirmation that the limbus remained viable even after AAV application. Furthermore, we would not have been able to observe a centripetal migration of GFP + cells in the limbal compartment towards central cornea had the limbus not remained viable throughout the duration of the experiment. Injection of different AAV serotypes is also necessary to compare the specific tropism of other serotypes and confirm whether AAV DJ is indeed the best candidate for transducing cells in human limbal region in situ. Further study is necessary to determine the optimal dosage as well as number of injections required to achieve the maximal transduction efficiency while minimizing potential toxicity. Future investigation is also required to confirm whether our injection technique specifically targets LSCs. This can be tested by comparing eGFP expression against a panel of other putative limbal stem cell markers (e.g., N-cadherin, ABCB5), as well as by testing the longevity and robustness of eGFP positivity in the stem cell compartment. Subsequent studies should also involve quantification of transduction efficiency and explore possible measures to improve the transduction efficiency.

In conclusion, our results show that cells in human limbal region can be targeted in situ using our sub-limbal injection technique in a reproducible manner, and AAV DJ is able to transduce these cells efficiently in situ. This technique may present a potential means of delivery for AAV-mediated gene therapy for inherited corneal diseases.

## Materials and methods

All injections were performed under a Nikon SMZ800 dissection microscope (Nikon, Melville, NY, USA).

### Ethics declarations

This study was approved by the Johns Hopkins Medicine Institutional Review Board (IRB00140342) and adhered to the tenets of the Declaration of Helsinki. Informed consent was obtained from all subjects and/or their legal guardian(s) by regional tissue eye banks.

### Human cadaver corneal tissues

Human cadaver corneoscleral tissues were procured after Descemet stripping automated endothelial keratoplasty (DSAEK) and Descemet membrane endothelial keratoplasty (DMEK) surgeries. After surgery, the remnant tissues were collected in shipping chambers containing Optisol-GS (Bausch & Lomb Inc., Bridgewater, NJ, USA) and transported to the laboratory. Trephined post-DSAEK corneal rims were used for trypan blue (0.4%, Sigma-Aldrich, Inc., St. Louis, MO, USA) and black India ink (Chartpak, Inc., Leeds, MA, USA) injections, while non-trephined post-DMEK tissues were used for AAV injections. A total of three corneas were used for each dye.

### Trypan blue and black India ink injection

Trephined corneal tissue was placed and secured on an artificial anterior chamber (Katena, Parsippany, NJ, USA) (Supplementary Fig. 1, 1A). Using 0.12 mm Colibri forceps (Storz Ophthalmic Instruments/Bausch & Lomb Corporation, Vaughan, CA), the conjunctival tissue overlying the limbal area (Supplementary Fig. 1, 1B, asterisk) was reflected to expose the limbus (Supplementary Fig. 1, 1B, arrowhead). 10-µl trypan blue or 10-µl black India ink was injected into the limbus by using a beveled 34-gauge needle (JBP Nanoneedle, South Korea) and a 1-ml syringe (BD Syringe, Franklin Lakes, NJ, USA) (Supplementary Fig. 1, 1C,D). The corneal tissue was then transferred to a humidified environment in a sterile 6-well cell culture plate containing one sheet of folded 2″ × 2″ sterile gauze soaked in 2 ml of phosphate-buffered saline (PBS; Quality Biological, Gaithersburg, MD, USA). It was left undisturbed at room temperature for 4 h before undergoing histological processing (see *Histology* below). Supplementary Video 1 demonstrates an exemplary video of trypan blue injection.

### AAV injection

Post-DMEK tissue was mounted on the artificial anterior chamber (Supplementary Fig. 1, 2A). Using 0.12 mm Colibri forceps, the conjunctival flap overhanging the limbal region (Supplementary Fig. 1, 2B, asterisk) was elevated and reflected to expose the limbus (Supplementary Fig. 1, 2B, arrowhead). AAV serotype DJ (Vigene Biosciences, Rockville, MD, USA) containing 10^10^ GC in PBS and harboring eGFP gene was injected into the sub-limbal region (Supplementary Fig. 1, 2C,D). Supplementary Video 2 shows an exemplary video of AAV injection.

The corneal tissue was then processed in the same manner as described above before undergoing corneal culture (see *Corneal Culture below*). AAV injection was repeated a total of three times on three consecutive days (Days 1–3). An overview of the AAV-DJ injection experiment is shown in Supplementary Fig. 1, panel 3.

### Corneal culture

For corneal tissues injected with AAV, the “air-lift” method was used to culture the corneal epithelium ex vivo as described in detail by Kramerov and colleagues^35^. In short, the AAV-injected corneal tissue was transferred to a sterile laminar flow hood and placed onto a sterile 60 mm petri dish (Corning Incorporated, Corning, NY, USA) with its epithelial side facing down. Corneal concavity was filled with 0.5 ml of gel composed of serum-free minimum essential medium (MEM; Thermo Fisher Scientific, Waltham, MA, USA), antibiotic–antimycotic (ABAM; Sigma-Aldrich, Inc), and 1 mg/ml calf skin collagen type 1 (MP Biomedicals, LLC., Solon, OH, USA) made from a stock solution of 10 mg/ml in 0.1 N acetic acid, 1% agar (Thermo Fisher Scientific), and insulin-transferrin-selenite supplement (InVitria, Junction City, KS, USA). After the cornea cavity gel solidified, the cornea was transferred to a sterile 6-well plate with its epithelial side facing up. Corneal medium was added to keep its level at the limbus for air–liquid interface culture, exposing the epithelium to air. The 6-well plate with the corneal tissue was placed in a humidified incubator with 5% CO_2_ at 35 °C. 150 μl of corneal medium was added onto the epithelium daily to moisten the corneas and the medium was changed every 2–3 days.

Corneas were processed for histological analyses on days 4 and 14 (since the date of the first AAV DJ-eGFP injection).

### Monitoring of epithelial proliferation

Epithelial proliferation was monitored at baseline, 4 days, and 7 days by instilling fluorescein dye onto the epithelium and illuminating with blue cobalt light under the microscope. After two weeks of incubation in air-lift culture, the tissues were processed for histological analyses.

### Histology

Tissues were fixed in 4% paraformaldehyde for 24 h at 4 °C, paraffin embedded and sectioned at 10-µm-thickness at the Johns Hopkins Hospital Pathology Laboratory.

### Immunohistochemistry

Paraffin sections were deparaffinized with xylene, rehydrated through a series of descending ethanol concentrations and washed in distilled water. Antigen retrieval was performed at sub-boiling temperature for 20 min using citric acid-based antigen unmasking solution (pH 6.0; Vector Laboratories, Inc., Burlingame, CA, USA), followed by permeabilization using 0.1% Triton X-100 (Sigma-Aldrich, Inc.) in PBS for 5 min. The sections were blocked in blocking serum containing 5 ml PBS with 3% bovine serum albumin (BSA) (9998S, Cell Signaling Technology, Inc., Danvers, MA, USA) and 2% goat serum (Sigma-Aldrich, Inc.) for 1 h. Primary antibodies diluted in blocking serum were added: rabbit anti-p63α (1:500 dilution) (Catalog #13,109; Cell Signaling Technology), chicken anti-GFP (1:100) (Catalog #A10262; Thermo Fisher Scientific), mouse anti-KRT19 (1:100) (Catalog #AB7754; Abcam, Cambridge, UK), and mouse anti-KRT3 (1:500) (Catalog #MA1-5763; Thermo Fisher Scientific), and the sections were incubated at 4 °C overnight. After rinsing 3 × 15 min with 0.01% Tween-20 (Sigma-Aldrich, Inc.) in PBS, secondary antibodies diluted in blocking serum were added: goat anti-rabbit IgG Alexa Fluor™ Plus 568 (1:500) (Catalog #A11036; Thermo Fisher Scientific), goat anti-chicken IgY Alexa Fluor™ 488 (1:500) (Catalog #A11039; Thermo Fisher Scientific), and goat anti-mouse IgG Alexa Fluor™ Plus 647 (1:500) (Catalog #A32728, Thermo Fisher Scientific), and the sections were incubated at room temperature for 1 h. After rinsing 3 × 15 min with 0.01% Tween-20 in PBS, 4′,6-diamidino-2-phenylindole (DAPI) (Catalog # D1306; Thermo Fisher Scientific) diluted in PBS (1:2000) was added for 3 min. Subsequently, the sections were washed 2 × 5 min with PBS and mounted with ProLong™ Diamond antifade mounting solution (Thermo Fisher Scientific).

### Microscopy

Light microscopy images of H&E slides were taken with Axio Imager.A2m (Carl Zeiss Microscopy LLC, White Plains, NY, USA). Immunohistochemical images were taken with a confocal light scanning microscopy (LSM) 710 (Carl Zeiss Meditec USA, Inc., Dublin, CA, USA).

## Supplementary Information

Supplementary Information 1.

Supplementary Information 2.

Supplementary Video 1.

Supplementary Video 2.

## Data Availability

The datasets generated during the current study are available from the corresponding author on reasonable request.
