# Supplementary figures and images for: In situ transduction of cells in human corneal limbus using adeno-associated viruses: an ex vivo study
Source: Sci Rep. 2022 Dec 28;12:22481. doi: 10.1038/s41598-022-26926-0 (PMC9797548; doi:10.1038/s41598-022-26926-0)

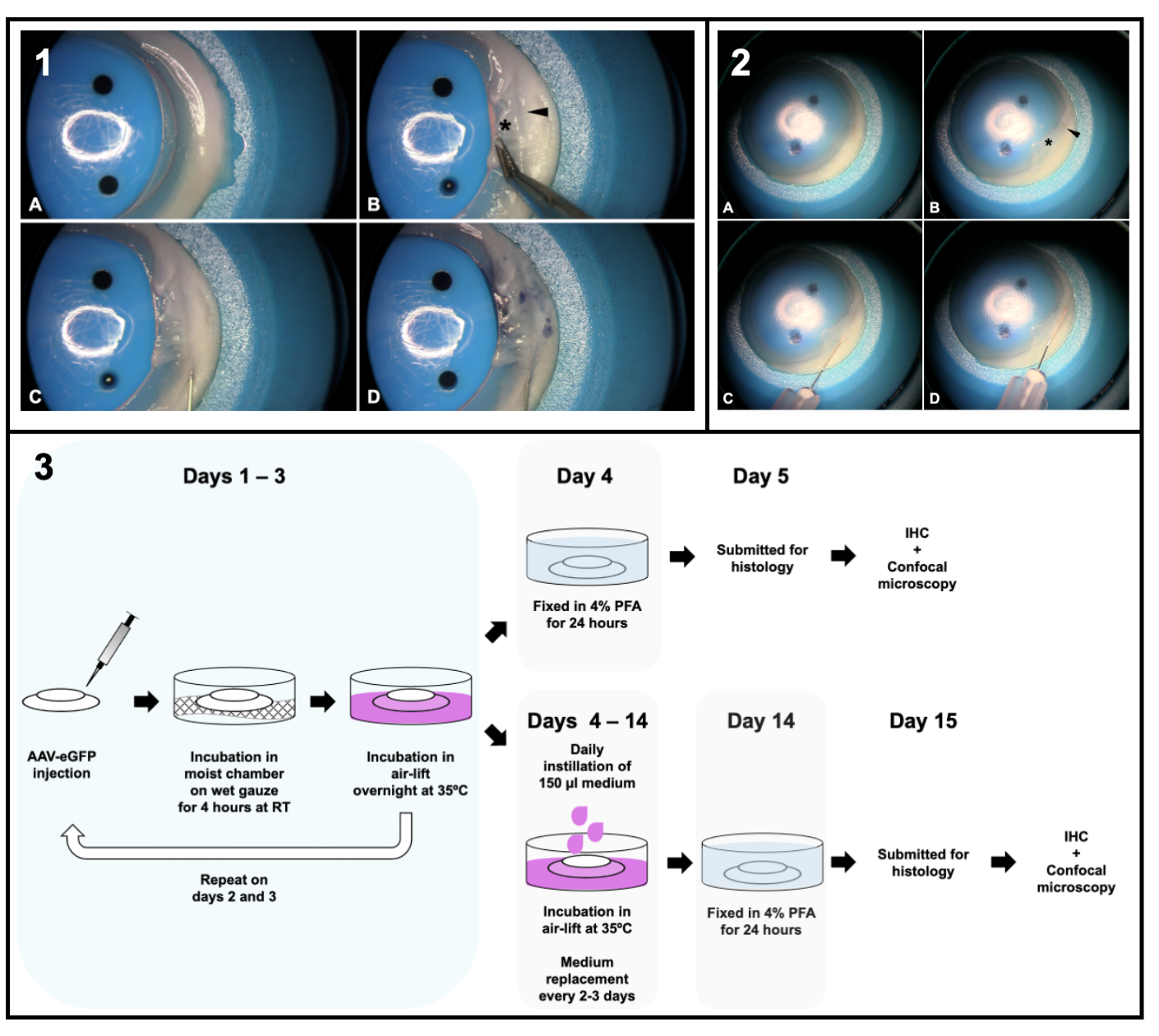

Supplement: Supplementary file 1 — Supplementary Information 1. [file 41598_2022_26926_MOESM1_ESM.tif]
