# Supplementary material for: In situ transduction of cells in human corneal limbus using adeno-associated viruses: an ex vivo study
Source: Sci Rep. 2022 Dec 28;12:22481. doi: 10.1038/s41598-022-26926-0 (PMC9797548; doi:10.1038/s41598-022-26926-0)
Supplement: Supplementary file 2 — Supplementary Information 2. [file 41598_2022_26926_MOESM2_ESM.docx]

**Supplementary Figure 1.** (**Panel 1**) Sub-limbal injection of a trephined human cadaveric corneoscleral rim previously used for Descemet Stripping Automated Endothelial Keratoplasty (DSAEK) surgery. **(1A)** The corneal tissue is placed and fixed on the artificial anterior chamber. **(1B)** Colibri forceps are used to elevate the conjunctiva (asterisk) and expose the limbal tissue (arrowhead). **(1C)** Angled 34G needle enters the limbal region with the bevel-side facing up. **(1D)** The needle is inserted gently and advanced tangentially along the limbus. Care is taken to stay as superficial as possible without perforating the tissue. Injection is performed approximately 1-2 clock hours away from the needle entrance site. (**Panel 2**) Sub-limbal injection of human cadaveric corneoscleral tissue previously used for Descemet Membrane Endothelial Keratoplasty (DMEK) surgery. **(2A)** The corneal tissue is placed and fixed on the artificial anterior chamber. **(2B)** Colibri forceps are used to reflect the conjunctiva (asterisk) and expose the limbal region (arrowhead). **(2C)** Angled 34G needle enters the limbal region with the bevel-side facing up. **(2D)** The needle is inserted gently and advanced tangentially along the limbus. Care is taken to stay as superficial as possible without perforating the tissue. Injection is performed approximately 1-2 clock hours away from the needle entrance site. (**Panel 3**) Experimental overview. On day 1, sub-limbal injection of AAV-GFP is followed by four hours of incubation in a humidified chamber at room temperature. Then, the corneal tissue is placed in 35°C incubator overnight in “air-lift”. The same steps are repeated on days 2 and 3. In two corneas, the tissues were fixed in 4% paraformaldehyde for 24 hours on day 4 and submitted for histology on day 5. In two other corneas, the tissues were incubated in air-lift culture for 14 days. 150 µl of corneal medium was pipetted onto the cornea daily between days 4 and 14 to moisturize the epithelial surface, and the medium was replaced every 2-3 days. On day 14, they were fixed in 4% paraformaldehyde for 24 hours before being sent to the pathology laboratory for processing on day 15. Immunohistochemistry was performed and the results were analyzed with a confocal microscope. *RT = room temperature; PFA = paraformaldehyde; IHC = immunohistochemistry.*

**Supplementary Video 1**. Sub-limbal injection of trypan blue into a trephined human cadaveric corneoscleral rim previously used for Descemet Stripping Automated Endothelial Keratoplasty (DSAEK) surgery.

**Supplementary Video 2**. Sub-limbal injection of adeno-associated virus serotype DJ diluted in phosphate-buffered saline into a human cadaveric corneal tissue previously used for Descemet Membrane Endothelial Keratoplasty (DMEK) surgery.
